# Refractory Coronary Vasospasm Leading to Cardiac Arrest: A Case Report

**DOI:** 10.7759/cureus.90692

**Published:** 2025-08-21

**Authors:** Ansh A Kamdar, Zane R Sink, Kartik Shatagopam

**Affiliations:** 1 Cardiovascular Medicine, Novant Health System, Salisbury, USA; 2 Internal Medicine, Carilion Clinic, Roanoke, USA; 3 Cardiovascular Medicine, Campbell University School of Osteopathic Medicine, Buies Creek, USA

**Keywords:** calcium channel blockers, cardiac arrest, clonidine, coronary vasospasm, icd, intracoronary nitroglycerin, prinzmetal's angina, refractory angina, vasospastic angina, ventricular fibrillation

## Abstract

We present a rare case of refractory coronary vasospasm in a 69-year-old African American male patient, initially misdiagnosed as typical thrombotic acute coronary syndrome (ACS) and treated with placement of a drug-eluting stent. The patient, unfortunately, experienced multiple subsequent readmissions and repeat angiography procedures due to refractory, multivessel vasospasm episodes despite compliance with medical therapy. This ultimately culminated in witnessed ventricular fibrillation arrest, requiring placement of an implantable cardioverter-defibrillator (ICD). This case underscores the diagnostic complexity of vasospastic angina, especially when it mimics obstructive coronary disease and ACS on both clinical presentation and angiography. Long-term management includes appropriate medication titration, patient education on symptom awareness and prompt treatment, and trigger avoidance. Six months post-discharge, the patient reported significant symptomatic improvement, no further hospitalizations, or ICD shocks. This case illustrates the potential severity of multivessel coronary vasospasm and supports a high index of suspicion in cases of unexplained recurrent chest pain, even following successful percutaneous coronary intervention (PCI).

## Introduction

Coronary vasospasm (or Prinzmetal's angina) refers to a transient, reversible constriction of the coronary arteries due to hyperreactivity of vascular smooth muscle. This variant form of angina was initially described by Dr. Myron Prinzmetal in 1959 [1]. The exact prevalence of vasospastic angina is poorly defined but appears to be more common in patients between the ages of 40 and 70 and higher in Asian populations than Caucasians [2].

While traditionally viewed as a benign condition, coronary vasospasm can sometimes mimic thrombotic acute coronary syndrome (ACS) both clinically and angiographically, leading to misdiagnosis and unnecessary percutaneous coronary intervention (PCI) [3]. The pathophysiology of coronary vasospasm is multifactorial, involving endothelial dysfunction, heightened vascular smooth muscle contractility, autonomic nervous system imbalance, and sometimes hypersensitivity to certain drugs or stimuli [4]. Although vasospastic angina often responds to calcium channel blockers and nitrates, some cases may become refractory and predispose patients to life-threatening ventricular arrhythmias or sudden cardiac death [5]. Furthermore, data demonstrate that vasospastic angina can contribute significantly to recurrent hospitalizations and adverse cardiac events in certain populations [6]. Consensus guidelines have now emphasized the need for diagnostic standardization to improve recognition and outcomes [7].

More routine use of intracoronary vasodilators during angiography and prior to PCI may prevent misdiagnosis by unmasking dynamic coronary narrowings mistaken for atherosclerotic or thrombotic lesions. In rare severe cases, including those with cardiac arrest, implantable cardioverter-defibrillator (ICD) therapy may be necessary for secondary prevention [8]. We present a unique case of refractory, multivessel coronary vasospasm initially misdiagnosed as thrombotic ACS, treated with drug-eluting stent placement. Our patient required multiple hospitalizations and repeat angiographies, culminating in ventricular fibrillation arrest and ICD placement. This case highlights the unique diagnostic and treatment challenges encountered in patients with refractory coronary vasospasm. 

## Case presentation

Index hospitalization

A 69-year-old African American male patient with past medical history of hypertension, type 2 diabetes mellitus, hyperlipidemia, chronic kidney disease, and prior tobacco use presented to the emergency department with several hours of more persistent substernal chest pain, preceded by three days of intermittent symptoms. On arrival, pertinent vital signs included blood pressure of 185/113 mmHg and pulse rate of 78 bpm. Echocardiogram (ECG) revealed new ST-segment depressions in inferior and lateral leads. Troponin T was significantly elevated at 44 ng/L.

**Figure 1:**
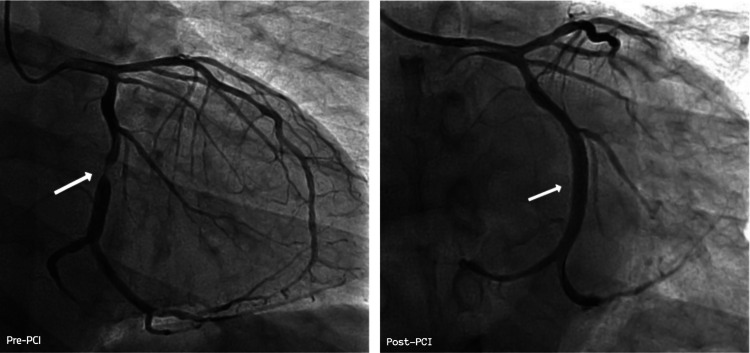
Initial coronary angiogram showing mid-circumflex artery lesion before and after drug-eluting stent placement

His chest pain resolved in the ER after the administration of sublingual nitroglycerin (SL NTG). He was admitted for ACS, started on a heparin drip, and monitored on cardiac telemetry overnight with no reports of recurrent chest pain. The patient underwent prompt coronary angiography the following morning, which demonstrated a 99% eccentric, thrombotic appearing narrowing in the mid-portion of the dominant circumflex artery, felt to be the culprit lesion. Given the typical clinical presentation of chest pain and ACS and a seemingly unambiguous culprit lesion on angiography, the patient proceeded directly to revascularization without further diagnostic imaging. The lesion was successfully treated with placement of a drug-eluting stent with excellent angiographic results (Figure 1). ECG revealed normal left ventricular wall motion with a normal ejection fraction of 60%. The patient had an unremarkable post-procedure hospital course with resolution of symptoms and ECG changes and was discharged the following day on guideline-directed medical therapy including dual antiplatelet therapy (aspirin + prasugrel), high-intensity statin, beta-blocker, and SL NTG for as-needed (prn) use.

Second hospitalization (recurrent chest pain)

**Figure 2:**
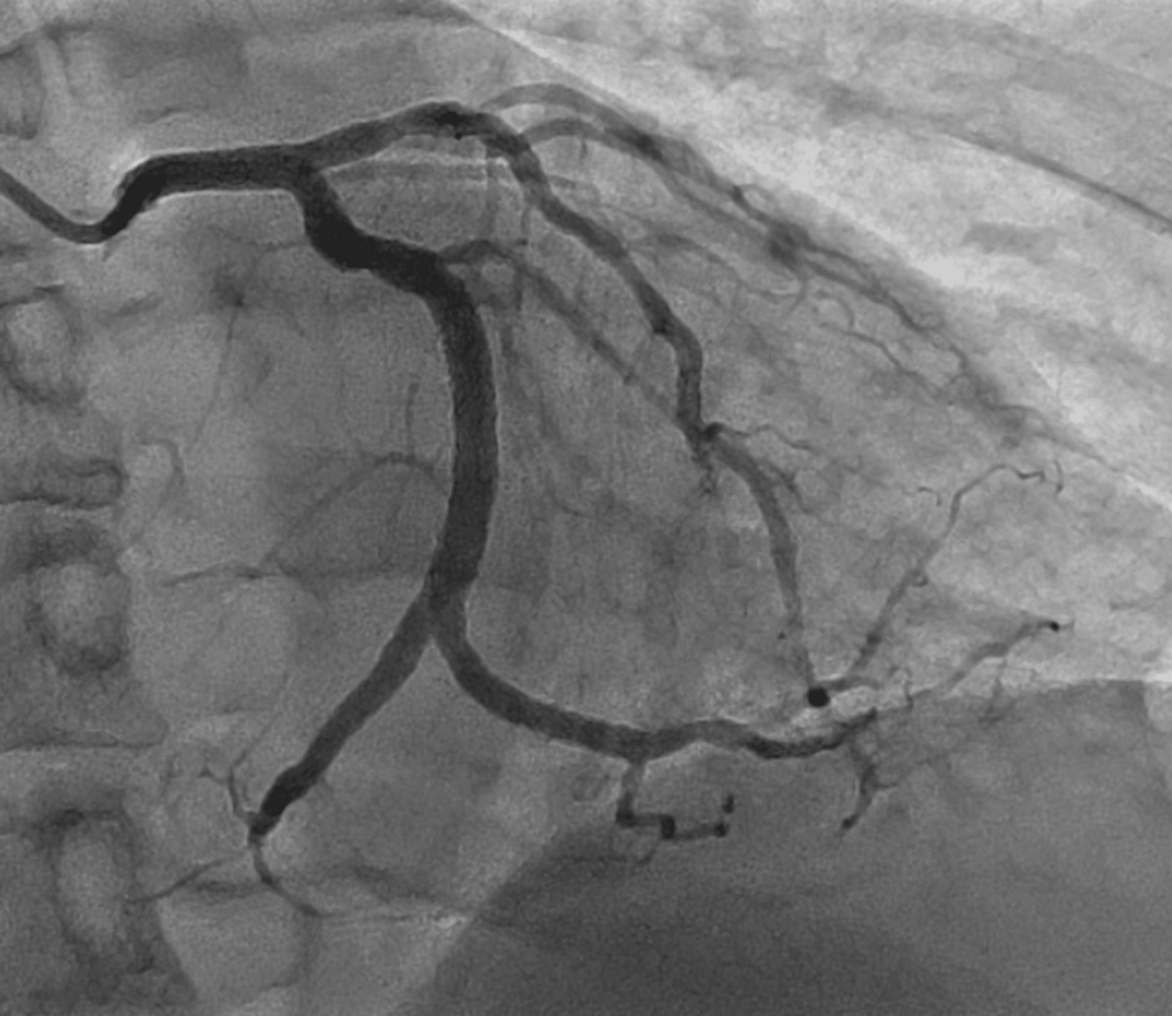
Repeat angiography demonstrating widely patent mid-circumflex stent

Four days post-discharge, the patient returned to the ER with recurrent, rest-onset chest pain. ECG showed recurrent inferior and lateral ST depressions. The patient confirmed compliance with all medications, including antiplatelet agents. The toxicology screen was normal. Repeat urgent coronary angiography and intravascular ultrasound (IVUS) showed a widely patent mid-circumflex stent that was well apposed with no evidence of in-stent thrombosis, stent edge disruption, or clear etiology to explain his pain (Figure 2). The patient was noted to be somewhat anxious, and chest pain was attributed to atypical, post-PCI chest pain and anxiety. As the patient's symptoms quickly resolved and no abnormalities were found, he was reassured and discharged on the same medical regimen with a plan for close outpatient follow-up.

Third hospitalization (vasospasm diagnosed)

**Figure 3:**
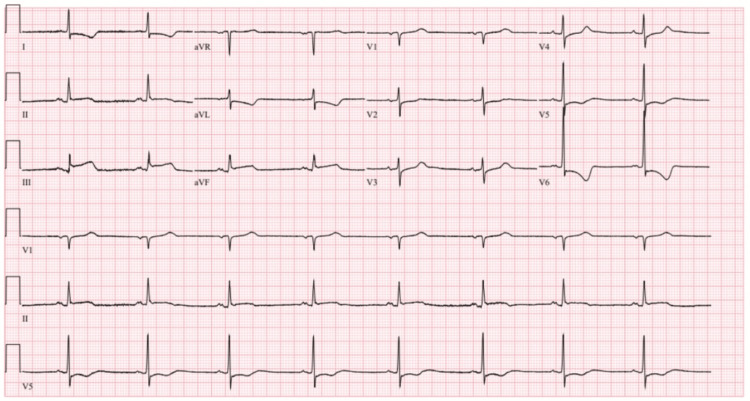
ECG showing ST-elevation myocardial infarction (STEMI) pattern with inferior ST elevations and reciprocal lateral depressions.

**Figure 4:**
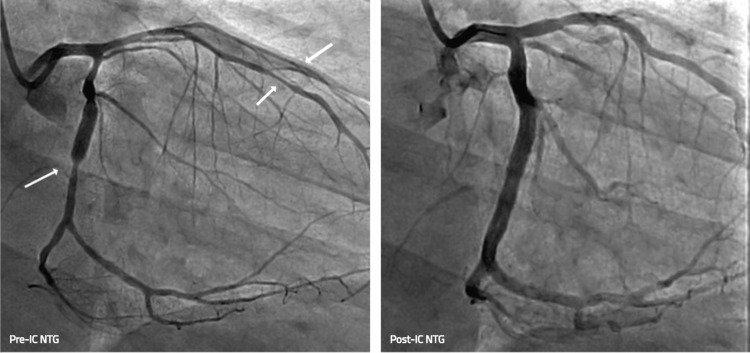
Angiographic images before and after intracoronary vasodilator administration demonstrating resolution of multivessel spasm of circumflex, LAD, and diagonal vessels LAD: left anterior descending artery

Six days later, the patient presented with severe nocturnal chest pain accompanied by nausea, diaphoresis, bradycardia, and hypotension. ECG now showed inferior ST-segment elevations and reciprocal high lateral/lateral depressions (Figure 3). Emergent repeat coronary angiography demonstrated diffuse multivessel coronary spasm involving the mid-left anterior descending artery (LAD), diagonal, and circumflex arteries, including marked focal spasm at the distal stent edge. Spasm resolved promptly after the administration of intracoronary (IC) NTG and nicardipine (Figure 4).

Having now established the diagnosis of coronary vasospasm, the patient was started on high-dose nifedipine and isosorbide mononitrate in addition to beta-blocker therapy, observed for clinical improvement, and subsequently discharged with counseling to continue to closely monitor symptoms, appropriate use of SL NTG, and to avoid typical triggers such as stress and secondhand tobacco exposure.

Fourth hospitalization (cardiac arrest)

**Figure 5:**
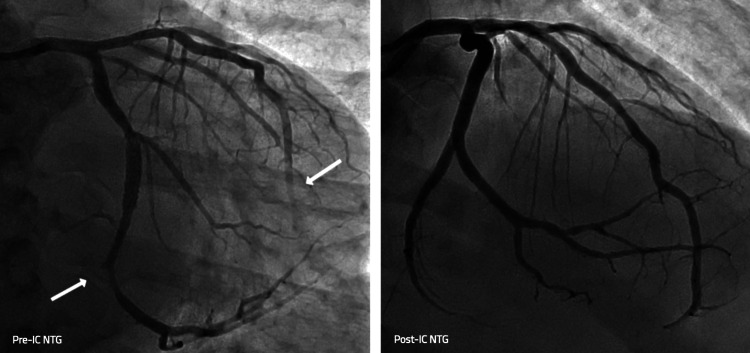
Coronary angiogram before and after intracoronary nitroglycerin during cardiac arrest event, demonstrating resolution of severe vasospasm of distal circumflex and mid LAD vessels LAD: left anterior descending artery

Four days later, the patient unfortunately presented yet again with severe chest pain, diaphoresis, and nausea. While being evaluated in the emergency department, he suddenly became unresponsive and was noted to be pulseless with seizure-like shaking. Telemetry confirmed ventricular fibrillation. CPR was briefly initiated, and the patient was rapidly defibrillated with a 120 J biphasic shock with prompt return of spontaneous circulation (ROSC). Post-arrest ECG showed deep T-wave inversions in the lateral leads. The patient underwent urgent repeat angiography, demonstrating severe multivessel coronary spasm, notably with TIMI Grade 1 flow in the mid and distal LAD and angiographic “cutoff” of the distal circumflex artery with TIMI Grade 0 flow. Again, the spasm responded readily to IC NTG (Figure 5). Post-procedure, the patient was admitted to the cardiac intensive care unit. Repeat ECG showed no significant wall motion abnormalities and preserved ejection fraction. Given the cardiac arrest secondary to refractory coronary vasospasm, he underwent dual-chamber ICD insertion for secondary prevention during the same hospitalization. His medical regimen was adjusted to include verapamil (in place of nifedipine), high-dose isosorbide mononitrate, and clonidine. Betablocker was discontinued.

Long-term follow-up

Over the subsequent six months, the patient reported several mild, transient episodes of chest discomfort responding to SL NTG but no further hospitalizations or ICD shocks. His symptoms improved significantly following medication adjustment and extensive discussion on the importance of early recognition and prompt self-treatment of anginal symptoms with SL NTG.

## Discussion

Coronary vasospasm remains a diagnostic challenge as it can closely mimic thrombotic ACS both clinically and angiographically [3]. Recurrent hospitalizations and repeated cardiac procedures in these patients can, unfortunately, also contribute to increasing their morbidity and complications [8].

In our case, the initial lesion in the mid-circumflex artery was presumed to be a thrombotic culprit lesion but was retrospectively likely due to dynamic coronary vasospasm. The diagnosis of vasospastic angina is challenging and often suspected or considered in the differential only after patients present with recurrent episodes of chest pain. Once the diagnosis was made in our patient, on more detailed and specific questioning, the patient did admit to prior episodic chest pains that fit with vasospastic angina; however, his chest pain certainly accelerated in both severity and frequency after coronary stent placement.

Stenting in vasospasm-prone patients can worsen vasospastic angina, felt to be triggered by localized endothelial irritation [5,9]. The routine use of intracoronary vasodilators such as NTG prior to PCI may help differentiate true fixed stenosis from dynamic coronary spasm and thereby prevent unnecessary stent placement.

The management of refractory coronary vasospasm includes aggressive medication titration with long-acting nitrates, calcium channel blockers, and alpha-agonists like clonidine. Beta-blockers have been described to sometimes worsen vasospasm, thought to be secondary to unopposed alpha-adrenergic activity, and although not absolutely contraindicated, may be avoided in refractory cases [10,11]. Both patient education and lifestyle modification are essential to manage and minimize recurrent events.

Our patient, unfortunately, experienced cardiac arrest due to refractory spasm despite successful diagnosis and adequate medical therapy. In such cases, ICD implantation is indicated for secondary prevention [8]. Long-term management requires ongoing medication titration, education, and lifestyle counseling to prevent recurrence.

Fortunately, on the most recent outpatient follow-up, our patient has been doing very well with minimal episodes of vasospastic angina on medical therapy and no recurrent hospitalizations, procedures, or ICD shocks.

## Conclusions

Although coronary vasospasm is typically considered a benign and reversible condition, this case illustrates its potential to mimic obstructive coronary artery disease and even result in life-threatening complications such as sudden cardiac arrest. A high index of suspicion, careful clinical history, and more routine use of intracoronary vasodilators may help avoid unnecessary coronary stenting. In severe cases with malignant arrhythmias, ICD placement may be warranted for secondary prevention. Aggressive medical therapy and patient counseling regarding early symptom recognition, prompt self-treatment, and trigger avoidance can improve outcomes in patients with refractory coronary vasospasm.
